# The carrot monoterpene synthase gene cluster on chromosome 4 harbours genes encoding flavour-associated sabinene synthases

**DOI:** 10.1038/s41438-020-00412-y

**Published:** 2020-12-01

**Authors:** Sven Reichardt, Holger Budahn, Dominic Lamprecht, David Riewe, Detlef Ulrich, Frank Dunemann, Lilya Kopertekh

**Affiliations:** Julius Kühn-Institut (JKI) - Federal Research Centre for Cultivated Plants, D-06484 Quedlinburg, Germany

**Keywords:** Plant breeding, Secondary metabolism

## Abstract

In plants, low molecular weight terpenes produced by terpene synthases (TPS) contribute to multiple ecologically and economically important traits. The present study investigates a carrot terpene synthase gene cluster on chromosome 4 associated with volatile monoterpene production. Two carrot mutants, *yellow* and *cola*, which are contrasting in the content of low molecular weight terpenes, were crossed to develop an F_2_ mapping population. The mapping analysis revealed overlapping QTLs on chromosome 4 for sabinene, α-thujene, α-terpinene, γ-terpinene, terpinen-4-ol and 4-carene. The genomic region of this locus includes a cluster of five terpene synthase genes (*DcTPS04*, *DcTPS26, DcTPS27, DcTPS54* and *DcTPS55*). *DcTPS04* and *DcTPS54* displayed genotype- and tissue-specific variation in gene expression. Based on the QTL mapping results and the gene expression patterns, *DcTPS04* and *DcTPS54* were selected for functional characterization. In vitro enzyme assays showed that *Dc*TPS54 is a single-product enzyme catalysing the formation of sabinene, whereas *Dc*TPS04 is a multiple-product terpene synthase producing α-terpineol as a major product and four additional products including sabinene, β-limonene, β-pinene and myrcene. Furthermore, we developed a functional molecular marker that could discriminate carrot genotypes with different sabinene content in a set of 85 accessions.

## Introduction

The cultivated carrot (*Daucus carota* L.) is an important horticultural crop due to characteristic flavour, nutritional value and health benefits of the root^1^. In addition, the above-ground tissues of carrot plants are gaining popularity in modern nutrition^2^. With a current annual world production of more than 40 million tons and a total growing area of about 1.2 million hectares, it ranks among the top ten vegetable crops^3^. In the past decades, breeding efforts mainly focused on increasing yield and enhancing disease and pest resistance. Nowadays, to address the consumer’s demands, the improvement of nutritional and flavour quality traits were added to the list of breeding aims^3^.

Low molecular weight terpenes are involved in plant-to-plant communication and plant protection against abiotic and biotic stresses^4,5^. The biological role of low molecular weight terpenes in plant protection against insect, fungal and bacterial pathogens has been demonstrated in several studies^6,7^. For example, overexpression of the rice terpene synthase gene *OsTPS19* functioning as a (S)-limonene synthase *in planta* lead to enhanced resistance to the blast fungus *Magnaporthe oryzae*^8^. In another report, it was shown that (E,E)-α-farnesene production is an important factor involved in pathogenesis of three post-harvest fungal pathogens (*Colletotrichum acutatum*, *Penicillium expansum* and *Neofabraea alba*) in apple fruit^9^. Furthermore, γ-terpinene displayed antibacterial activity against *Xanthomonas oryzae* in rice^10^.

Moreover, relevant to this study, the low molecular weight terpenes contribute to carrot flavour^11^. The flavour combines taste and aroma and is a complex trait that depends on the relative amount of sugars, non-volatiles (amino acids, phosphates, polyacetylenes, nitrogenous substances) and volatiles (terpenes, phenylalanine- and fatty acid-derived compounds)^12^. The typical flavour of carrots has been attributed mainly to volatile terpenes, with mono- and sesquiterpenes representing approximately 98% of the volatile compounds^11,13^. Few studies have focused on the correlation between terpenoid volatiles and carrot sensory attributes^14–16^. Because carrots contain a complex blend of many different terpene compounds, it is challenging to relate single terpenes to specific aroma characteristics^13^. Using an GC-olfactometry (GC-O) approach, a link between the carrot aroma and flavour and certain isolated terpenes could be established^13^. Odour sensation notes such as “carrot top”, “terpene-like”, “green”, “fruity”, “spicy” or “woody” were defined as odour descriptors. The monoterpenes sabinene, β-myrcene and p-cymene were reported to be important contributors to the “carrot top” aroma, whereas limonene, γ-terpinene and terpinolene are related to fruity notes. Sesquiterpenes like β-caryophyllene, α-humulene and γ-bisabolene contributed to “spicy” and “woody” notes^13^. Among some non-volatile compounds like polyacetylenes or laserine derivatives terpenes are often involved in harsh or bitter taste^15^. A combination of a chemical and a sensorial approach predicted sabinene, α-terpinolene and β-pinene as candidates for bitterness^5^.

The huge variety of different terpenes is formed by members of terpene synthases family (TPS, EC 4.2.3) from few substrates by similar carbocation-based reaction mechanisms^17,18^. Thus, monoterpene synthases synthesize monoterpenes from the substrates geranyl diphosphate (GPP) and its *cis*-isomer neryl diphosphate (NPP); sesquiterpene synthases from sesquiterpenes from both isomer forms farnesyl diphosphate (*trans*-FPP and *cis*-FPP). In this reaction, the initial ionization of the substrates results in a cationic intermediate, which undergoes a series of rearrangements, until the reaction is terminated by a proton loss or addition of a nucleophile^19,20^. The family of terpene synthases has been divided into eight clades (TPS-a to TPS-h) based on sequence properties^21^. In the carrot genome, 65 putative terpene synthase genes have been identified. Most of them belong to the clades TPS-a and TPS-b^22,23^.

Several studies are focusing on the genomic regions and genes potentially involved in synthesis of carrot terpenes including flavour-associated terpenes. Based on a genome-wide association study (GWAS), 21 QTLs have been described for 11 terpenes in roots^23^. Clusters of terpene synthase genes have been found on chromosomes 3, 4, 5, 7 and 9. A recent study, which focused on bitterness trait variation in a carrot F_2:3_ population, identified 71 QTLs for 25 terpenes^5^. These QTLs were distributed on chromosomes 3, 4 and 9. Both studies described major QTLs for low molecular weight terpenes on chromosome 4. However, the map-based cloning and further characterization of candidate genes has not been performed yet. Two studies have reported on functional characterization of terpene synthases from carrot. The first study presented data on an in vitro test for *Dc*TPS1 and *Dc*TPS2 belonging to the TPS-a and TPS-b subfamilies, respectively. Recombinant *Dc*TPS1 produced the sesquiterpenes (E)-β-caryophyllene and α-humulene, while *Dc*TPS2 synthesized the monoterpenes geraniol and β-myrcene^24^. Another work showed that a monoterpene synthase (*WtDc*TPS1) from a wild carrot produced geraniol and β-myrcene in an in vitro assay^25^.

The utilization of molecular markers linked to QTLs can support modern carrot breeding. The application of molecular markers can help to select putative crossing parents by analysis of functional allelic diversity of *Daucus* germplasm and to screen the progenies for desired gene combinations. Marker-assisted selection in carrot has been performed for resistance against the root-knot nematode *Meloidogyne javanica*^26^ and root quality traits, particularly carotenoid and sugar composition^27^. However, no functional molecular markers that might be used for breeding of different terpene profiles in carrot genotypes have been reported yet.

In this study, we investigated a terpene synthase gene cluster on carrot chromosome 4. To define the genomic regions associated with terpene production, a QTL analysis followed by expression analysis of *DcTPS04*, *DcTPS26*, *DcTPS27*, Dc*TPS54* and *DcTPS55* genes was performed. Finally, the Dc*TPS04* and Dc*TPS54* were functionally characterized in an in vitro assay.

## Results

### Phenotyping and QTL mapping in a F_2_ population

**Fig. 1:**
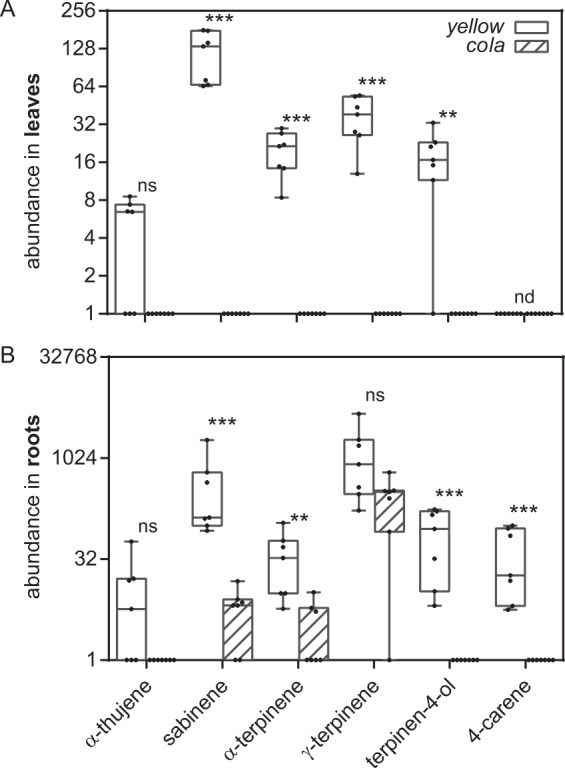
Abundance of monoterpenes in *yellow* and *cola* mutants. Leaf (**a**) and root (**b**) tissue of seven plants was analysed by headspace GC-MS. The results are presented in box and whisker plots (min to max, show all points) with a fold change of the abundance on a log2 scale. The data are statistically analysed with Mann–Whitney test (*P < 0.05, **P < 0.01, ***P < 0.001, not significant (ns) and not detectable (nd))

A prerequisite for QTL analysis is two parent genotypes that are different in the trait of interest. We observed that the *yellow* and the *cola* carrot genotypes display significant phenotypic differences in the content of low molecular weight terpenes. In comparison to the *yellow genotype*, the *cola* genotype showed reduced amounts of terpinen-4-ol, α-terpinene, sabinene in leaf and root tissue and γ-terpinene in leaves. The monoterpene 4-carene was detectable in roots of the *yellow* mutant but not in the leaves of this genotype (Fig. 1).

**Fig. 2:**
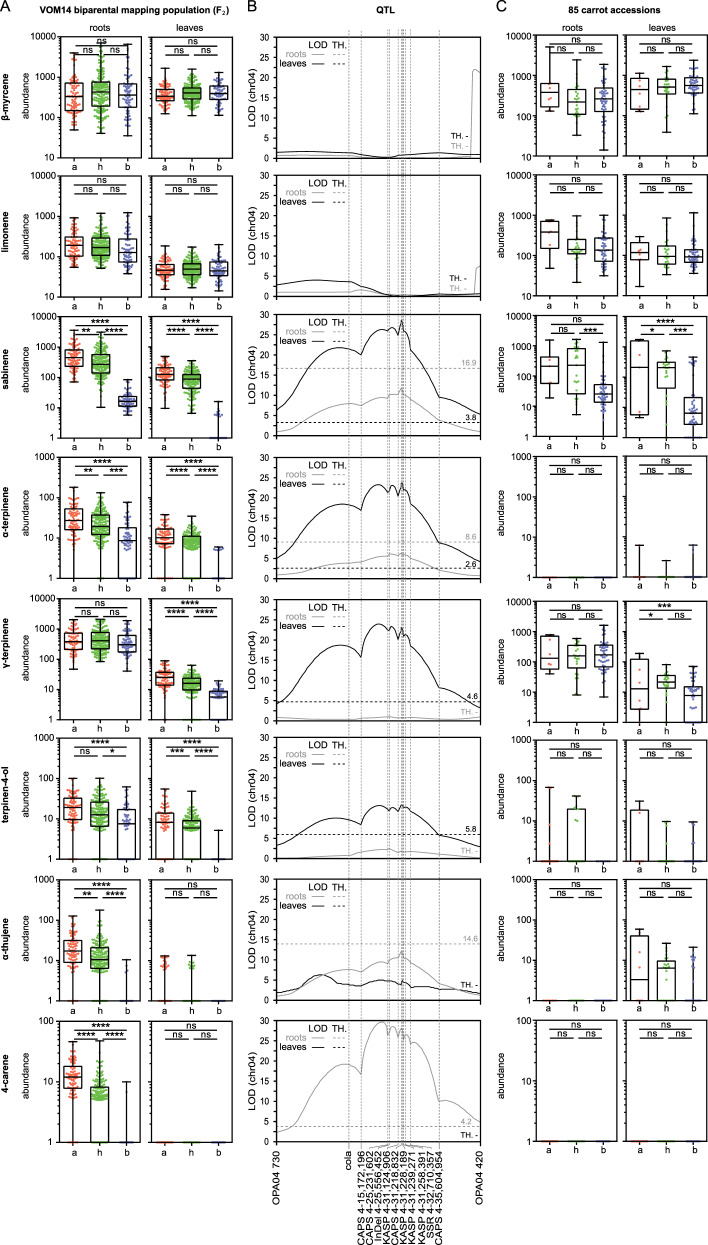
Marker dependent abundance of terpenes and LOD scores for chromosome 4. **a** Abundance of terpene in the VOM14 F_2_ biparental mapping population sorted after marker genotyping. Three hundred and twenty plants were analysed by headspace GC-MS and the terpene content was sorted by the results of the marker analysis (KASP 4-31,228,189 CAPS 4-31,218,832). Association consistencies between the abundance of β-myrcene, limonene, sabinene, α-terpinene, γ-terpinene, terpinen-4-ol, α-thujene and 4-carene, and genotype in leaf and root tissue of carrot are presented in box and whiskers (min to max, show all points) with a fold change of the abundance in log10 scale. The data were statistically analysed with Tukey’s multiple comparison test (*P < 0.05, **P < 0.01, ***P < 0.001, ****P < 0.0001, not significant (ns)). **b** LOD score of terpenes associated with chromosome 4 based on markers flanking the terpene synthase gene cluster. Significance threshold (TH) was set using 1.000 permutations. **c** Validation of the KASP 4-31,228,189 marker. KASP-based genotyping of 85 carrot accessions. Association consistency between the abundance of β-myrcene, limonene, sabinene, α-terpinene, γ-terpinene, terpinen-4-ol, α-thujene and 4-carene and genotype in leaf and root tissue of carrot. All measured points were statistically analysed by Tukey’s multiple comparison test (*P < 0.05, **P < 0.01, ***P < 0.001, ****P < 0.0001, not significant (ns)) and presented in box and whisker plots. The abundance is shown with a fold change on a log10 scale. The horizontal *x*-axis (**a**, **c**) indicates genotypic groups a, b and h. The *y*-axis (**a**, **c**) indicates an abundance of terpenes in investigated plants and is on a log10 scale. The plants in group a carry the homozygous maternal allele (**a**), whereas the plants in group b harbour the homozygous paternal allele (**b**). Individuals in group h are the heterozygous plants

These two contrasting parental genotypes were crossed to develop a QTL mapping population. The F_2_ generation of this population comprised 320 individual plants. They were analysed for volatile terpene compounds using headspace SPME-gas chromatography. This analysis allowed us to quantify 20 monoterpenes and 15 sesquiterpenes in root tissue, and 18 monoterpenes and 18 sesquiterpenes in leaf tissue, respectively (Tab. S1). In leaves, the most abundant monoterpenes were β-myrcene, sabinene and limonene, and the major sesquiterpenes were β-caryophyllene and β-bisabolene. The terpenes with the highest abundance in roots were the monoterpenes terpinolene, γ-terpinene and sabinene and the sesquiterpenes β-caryophyllene, guaia-3,9-diene and α-bisabolene (Tab. S1). Phenotypic variation in the contents of sabinene, α-terpinene and terpinen-4-ol (leaf and root tissue), 4-carene, α-thujene (root tissue) and γ-terpinene (leaf tissue) was observed in the F_2_ population. No significant differences could be measured for the non-circular compound β-myrcene and the circular limonene, functioning as unaffected controls (Fig. 2a).

Genotypic analysis was performed using 89 molecular markers that were polymorphic in the F_2_ mapping population. This set of molecular markers was distributed over all carrot chromosomes and included 33 SSR-, 13 CAPS-, 9 KASP-, 24 InDel- and 10 RAPD markers. Twenty-eight molecular markers close to TPS genes are presented in Tab. S2. Markers with segregation distortion were excluded from map construction. The resulting partial genetic map had a length of 536.6 cM encompassing all nine *Daucus carota* chromosomes (data not shown).

QTL analysis was conducted using phenotypic and genotypic data from the F_2_ mapping population. In total, 14 QTLs with LOD scores >10 were identified for terpenes in roots and leaves (Table 1).

**Table 1:** QTLs identified in the F_2_ mapping population (VOM14)

No.	Tissue	Chr.	Flanking markers	Target	LOD	PVE (%)
1	Leaf	3	*DcTPS53*_3-48.693.414	β-elemene	28.48	35.70
2	Leaf	3	*DcTPS53*_3-48.693.414	δ-elemene	26.60	34.10
3	Leaf	3	*DcTPS05*_3-45.438.469	β-farnesene	10.93	15.00
4	Leaf	4	*DcTPS54*_4-31.228.189	Sabinene	27.49	33.60
5	Leaf	4	*DcTPS04*_4-31.218.832	γ-terpinene	20.48	26.60
6	Leaf	4	*DcTPS54*_4-31.228.189	α-terpinene	20.37	26.10
7	Leaf	4	*DcTPS04*_4-31.218.832	Terpinene-4-ol	12.52	16.90
8	Root	1	*DcTPS10*_1-44.682.063	Bornyl acetate	12.86	17.30
9	Root	3	*DcTPS05*_3-45.438.469	Guaia-3,9-diene	30.76	36.50
10	Root	3	*DcTPS53*_3-48.693.414	δ-elemene	22.34	28.00
11	Root	4	*DcTPS54*_4-31.228.189	4-carene	28.68	34.30
12	Root	4	*DcTPS54*_4-31.228.189	α-thujene	12.57	16.80
13	Root	4	*DcTPS54*_4-31.228.189	Sabinene	10.11	13.70
14	Root	5	*DcTPS57*_5-29.660.476	β-pinene	15.52	21.30

In carrot roots, QTLs associated with bornyl acetate and β-pinene were detected on chromosomes 1 and 5, respectively. QTLs located on chromosome 3 were calculated for β-elemene, δ-elemene and β-farnesene in leaves and for guaia-3,9-diene and δ-elemene in roots. A genomic region spanning 105 kb on chromosome 4 includes QTLs for sabinene, α-thujene, α-terpinene, γ-terpinene, terpinen-4-ol and 4-caren. Five molecular markers, namely KASP 4-31,228,189, CAPS 4-31,218,832, KASP 4-31,147,906, KASP 4-31,258,391 and KASP 4-31,239,271 were associated within this region (Fig. 2b). These molecular markers segregated in the F_2_ mapping population according to the Mendel’s law of segregation (Fig. S1, Tab. S3). Highly significant QTLs were detected for sabinene (LOD: 27.49), α-terpinene (LOD: 20.37), γ-terpinene (LOD: 20.48) in leaves and for 4-carene (LOD: 28.68) in roots. Although the LOD scores for other analysed low molecular weight terpenes showed the same tendency, they did not exceed the significance threshold calculated individually for each terpene.

### Molecular markers associated with sabinene content

Genotyping of the F_2_ mapping population with the molecular markers KASP 4-31,228,189 and CAPS 4-31,218,832 allowed the identification of three groups ‘a’, ‘h’ and ‘b’ with different content of specific monoterpenes (Fig. 2a). The plants in these groups differed regarding the homozygous maternal allele (a), the homozygous paternal allele (b) and the heterozygous genotype (h). The highest average content of specific monoterpenes was always detected in group ‘a’ and the lowest in group ‘b’ (Fig. 2a).

To test the applicability of the molecular marker KASP 4-31,228,189, we investigated a panel of 85 carrot genotypes described previously^23^. The group ‘a’ harbouring genotypes, homozygous for the allele (a), consists of nine genotypes, including the *yellow* mutant. Fifty-one accessions and the *cola* mutant are arranged in group ‘b’, which is homozygous for allele (b). Twenty-five accessions are members of group ‘h’ displaying the heterozygous genotype (Fig. S3). As expected, marker-trait association analysis for the control substances β-myrcene and limonene showed no significant differences. For the sabinene content in leaf tissue a strong correlation was found. The homozygous alleles (a) and (b) were associated with high and low sabinene content, respectively. The same tendency was observed for sabinene in root tissue (Fig. 2c). For α-terpinene, terpinen-4-ol, and 4-carene no significant differences were observed, due to their low detectability. For γ-terpinene, the marker-trait association in leaves showed the same tendency as for sabinene (Fig. 2c). Our data show that the molecular marker KASP 4-31,228,189 can discriminate carrot genotypes by a specific allelic configuration for sabinene production. To validate the major QTL for sabinene, we investigated candidate genes with tentative terpene synthase activity within the terpene synthase gene cluster on chromosome 4.

### Terpene synthase cluster on chromosome 4 includes putative monoterpene synthases

**Fig. 3:**
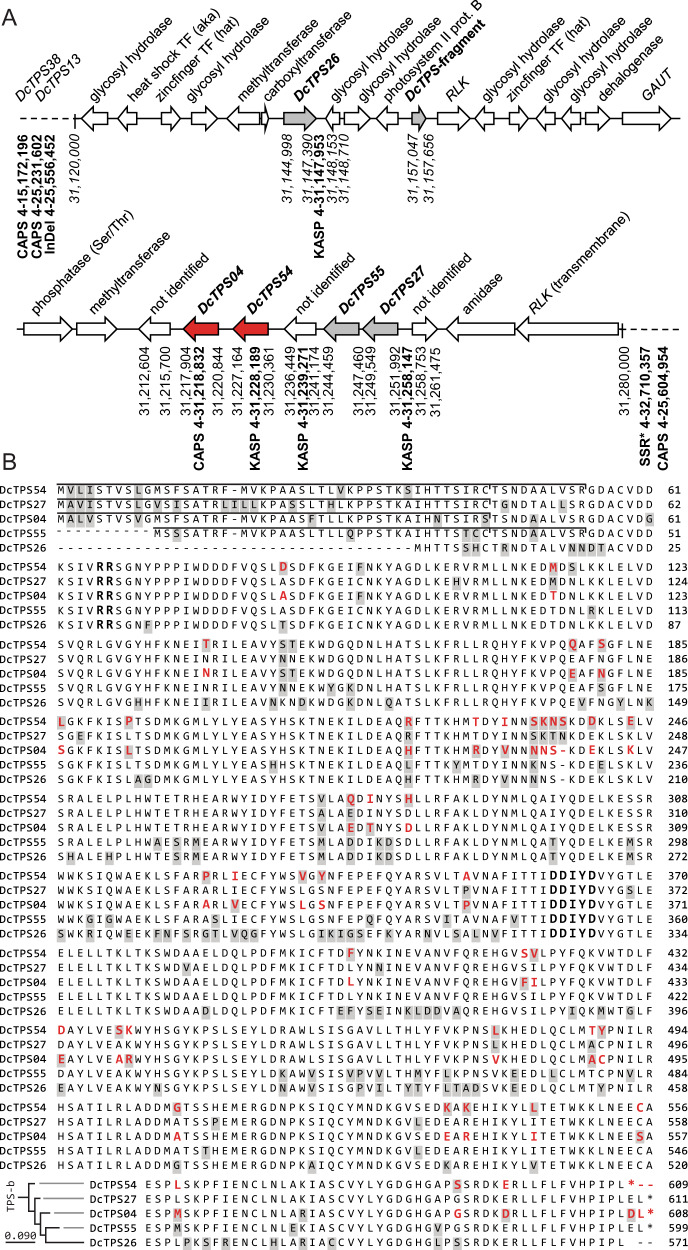
Characterization of a genomic region associated with sabinene production on chromosome 4. **a** Schematic representation of the TPS-gene cluster. Structural analysis of this region was performed based on reannotation of carrot genome assembly by GeMoMa (version 1.6.3.beta) using 12 reference genomes^22,28^. Receptor-like kinase (RLK) and galacturonosyltransferases (GAUT). **b** Amino acid sequence alignment of candidate terpene synthases from the *yellow* and *cola* mutant. Background in grey shows different residues in all five candidate terpene synthases. Differences between *DcTPS04* and *DcTPS54* sequences are marked in red. N-terminus of the catalytic domain (RR) and the DDIYD-conserved motif are marked in bold. The N-terminal plastid transit peptide predicted by Target P2.0 is marked with a horizontal line. The phylogram in the lower-left corner shows similarity of the catalytic domain of the 5 terpene synthases based on the translation of the nucleotide sequences from the *yellow* and the *cola* mutant. TPS-b marks the as TPS-b clade. A phylogenetic tree of all carrots TPS was previously published in ref. ^23^

For in silico analysis of the genomic region on chromosome 4 associated with sabinene production, we performed a reannotation of the carrot genome using 12 reference genomes in GeMoMa 1.6.2beta^22,28^. This gene cluster with the genomic coordinates 31,120,000–31,280,000 bp includes genes encoding proteins of different functional groups. Two zinc finger transcription factors, two methyltransferases, one phosphatase, one galacturonosyl transferase (GAUT), seven glycosyl hydrolases (GH) and five terpene synthases were predicted (Fig. 3a).

The putative glycosyl hydrolase genes are homologous to plant xyloglucan endotransglucosylases (XET). Out of 36 XET genes that were recognized in the carrot genome, seven XETs surround the *DcTPS26* gene.

We identified five terpene synthase genes, *DcTPS04* (DCAR_013298), *DcTPS26* (DCAR_013310), *DcTPS27* (DCAR_013293), *DcTPS54* (DCAR_013297) and *DcTPS55* (DCAR_013294) confirming the terpene synthase gene prediction reported previously^22,23^. The terpene synthase genes spread over a region of 105 kb. The genes *DcTPS04*, *DcTPS27, DcTPS54* and *DcTPS55* are located physically within a genomic region of 35 kb and genetically within 0.11 cM. The gene *DcTPS26* is located 70 kb upstream of *DcTPS04* (Fig. 3a). In addition, a 609 bp TPS-fragment at position 31,157,047 –31,157,656 bp was found. Its amino acid sequence has 86% identity to the first 150 residues of the N-terminus of *DcTPS26*. However, a large part of the catalytic domain is missing. Thus, it can be assumed that this terpene synthase gene fragment is non-functional. The amino acid sequence alignment of the five predicted terpene synthase genes revealed their high sequence similarity, varying from 78.5 to 91% (Fig. 3b). Based on high sequence similarity and chromosomal orientation of *DcTPS04*, *DcTPS26, DcTPS27, DcTPS54* and *DcTPS55*, the same evolutionary origin can be suggested. Although *DcTPS26* has a high sequence similarity to other monoterpene synthases, the lack of a proper transit peptide indicates a sesquiterpene producing activity (Fig. 3b, Fig. S3). Proper transit peptides were predicted by Target P2.0 algorithm for *Dc*TPS04 (the cleavage site position CS pos: 54–55, between the residues VSR-GD, with a prediction Pr. of 0.2131), for *Dc*TPS54 (CS pos: 44–45, IRC-TS. Pr: 0.7755), for *Dc*TPS55 (CS pos: 34–35, TCC-TS, Pr: 0.2387) and for *Dc*TPS27 (CS pos: 45–46, IRC-TG, Pr: 0.7556), indicating their monoterpene synthase activity.

### Expression pattern of terpene synthase candidates

**Fig. 4:**
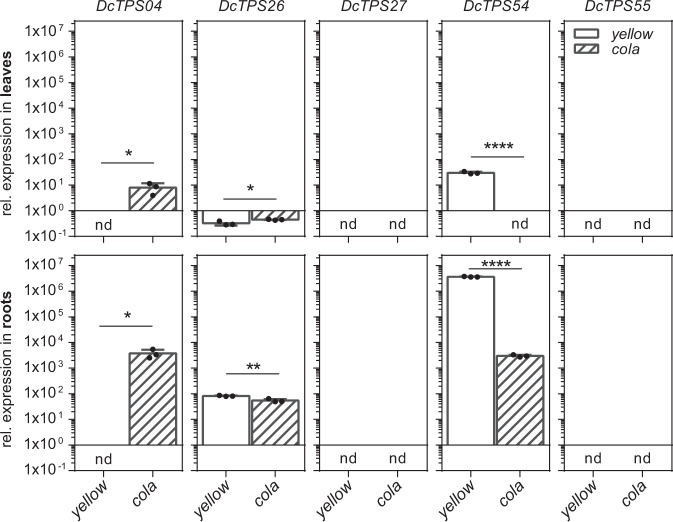
Expression profile of terpene synthases from gene cluster on chromosome 4. The levels of RNA transcripts for *DcTPS04*, *DcTPS26*, *DcTPS27*, *DcTPS54* and *DcTPS55* genes were analysed in roots (mix of cortex, xylem and phloem tissues) and leaves (mix of young leaf and petiole tissues). Each sample was pooled from three plants. Each experiment was performed in biological and technical triplicates. Relative expression for the five TPS gene in *yellow* and *cola* mutants was calculated using the Pfaffl method with the reference genes *DcTub*, *DcHSP, DcPP2A* and presented with a fold change in a log10 scale. Relative to *DcTub* expression levels are presented in plots with standard deviation. Data were statistically analysed by unpaired *t*-Test (*P < 0.05, **P < 0.01, ****P < 0.0001). Samples with non-detectable CTs for three biological replicates are marked as nd

We analysed the expression patterns of *DcTPS04*, *DcTPS26*, *DcTPS27*, *DcTPS54* and *DcTPS55* clustered on chromosome 4 to assign a priority to candidate genes, which could be involved in sabinene production. For this purpose, total RNA was isolated from leaf and root tissue of the two 16 weeks old carrot mutants (*cola* and *yellow*) and was analysed with gene-specific primers by qRT-PCR. Transcript accumulation was obtained for *DcTPS04*, *DcTPS26* and *DcTPS54*, whereas the transcripts of *DcTPS27* and *DcTPS55* genes could hardly be identified (Fig. 4).

Our results show that *DcTPS04, DcTPS26* and *DcTPS54* transcript levels are higher in root tissue compared to leaf tissue (Fig. 4). For *DcTPS26* we detected lower expression levels in leaf tissue of *yellow* compared to *cola*. In root tissue, the expression of *DcTPS26* was higher as in *cola*. Due to these contrasting expression patterns, *DcTPS26* was not selected for further analysis. We also observed differences in the expression of *DcTPS04* and *DcTPS54* in the *yellow* and *cola* mutant. The transcript of *DcTPS04* was detected in root and leaf tissue of *cola* and it was not detectable in *yellow*. In contrast, higher expression levels of *DcTPS54* were found in *yellow* compared to *cola* (Fig. 4). The expression profile of *DcTPS54* is in good agreement to the abundance of sabinene in *yellow* and *cola* mutants (Fig. 1). The expression pattern of *DcTPS04* and *DcTPS54* in *yellow* and *cola* gives an indication for their involvement in sabinene synthesis in these genotypes. Based on the gene expression and QTL analysis, we selected *DcTPS04* and *DcTPS54* for further functional analysis.

### Functional characterization of sabinene synthase candidates

**Fig. 5:**
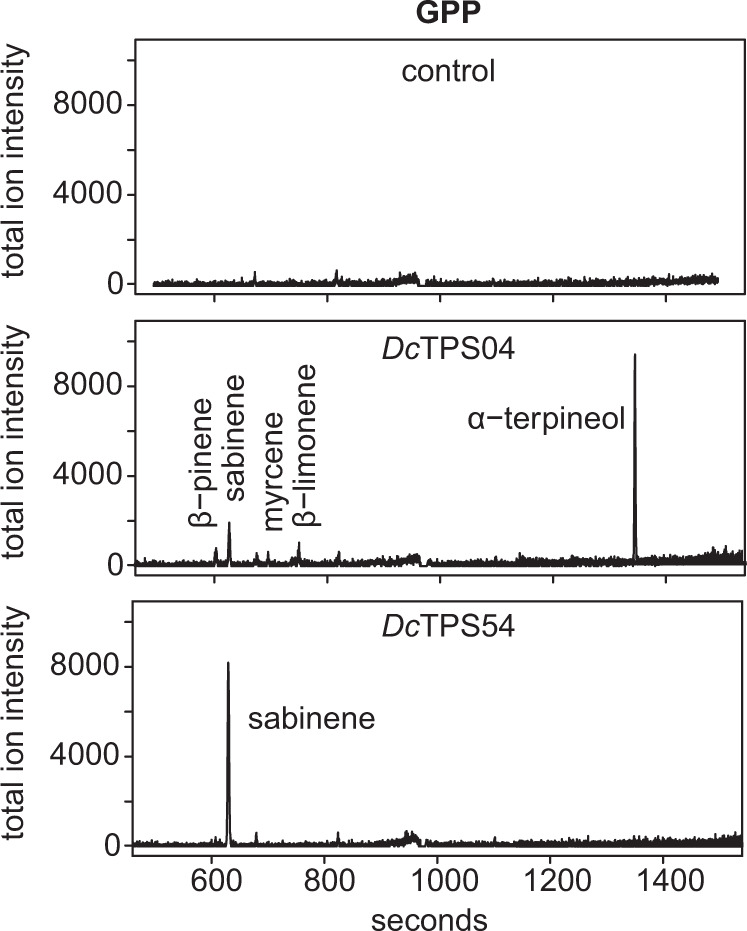
GC-MS of the products formed by the *DcTPS04* and *DcTPS54* in an in vitro assay. The *Dc*TPS04 and *Dc*TPS54 proteins were incubated with GPP substrate for 30 min and products were analysed by GC-MS. The data for NPP, (E,E)-FPP and (Z,Z)-FPP are presented in Fig. S4. The representative gas chromatograms for three individual experiments are shown

For functional characterization of *Dc*TPS04 and *Dc*TPS54, we cloned the ORF of the catalytic domains excluding the N-terminal region with the transit peptide. The amplified sequences of 1632 bp and 1692 bp for *DcTPS04* and *DcTPS54*, respectively, were integrated upstream of the 6-His tag and following stop codon in pET28c plasmid and expressed in *E. coli* Rosetta^TM^ 2 strain. The synthesised terpene synthases were assayed for monoterpene (GPP and NPP substrates) and sesquiterpene ((E,E)-FPP and (Z,Z)-FPP substrates) synthase activity. The products were analysed by mass spectrometry. Enzymatic formation of terpenes was observed only with GPP substrate identifying *Dc*TPS04 and *Dc*TPS54 as functional monoterpene synthases (Fig. 5, S4).

*Dc*TPS54 yielded the single monoterpene product sabinene. The major product of *Dc*TPS04 is α-terpineol along with the minor products sabinene, β-limonene, β-pinene and myrcene. Extracts prepared from *E. coli* served as controls for terpene formation in vitro. These extracts did not produce any detectable amounts of terpenes.

Our data clearly show that both proteins catalyse sabinene formation in vitro. Moreover, the functional test revealed *Dc*TPS54 as a single-product terpene synthase and *Dc*TPS04 as a multiple-product terpene synthase. Therefore, we were able to confirm the sabinene-associated QTL on chromosome 4.

## Discussion

In the present study, we investigated the genomic region on carrot chromosome 4 associated with monoterpene production by terpene metabolite profiling, QTL mapping and functional analysis of terpene synthase candidate genes. This combinatorial approach allowed us to identify terpene synthase genes responsible for sabinene synthesis.

To dissect the genetic basis of a quantitative trait, such as the content of a natural product, two main methods are available, conventional QTL mapping and GWAS. QTL mapping depends on genetic diversity of two parents and is time-consuming due to the necessity to develop a mapping population. Moreover, QTL regions can be quite large and may include many potential candidate genes. Nevertheless, this method has been used in carrot research and breeding to elucidate genetic control for morphological, disease resistance and root quality traits^3,29^. GWAS can overcome the limitations of QTL mapping. This technique has great potential for detection of QTLs with high resolution in diverse sequenced genotypes. However, GWAS also can have high false-positive rates due to the population structure of most germplasm sets^30,31^. GWAS addressing volatile compounds has already been conducted in studies applied to tomato, apple and blueberry^32–34^.

There are only few reports on the use of GWAS for QTL identification in carrot. This technique was used, to discover the *Or* gene on chromosome 3^35^ and to link volatile terpenes and their potential synthases^23^. A combination of QTL mapping and GWAS is a promising strategy to compensate the limitations of each method. However, a combined approach has not been performed in carrot, yet.

Our analysis of a F_2_ mapping population revealed 14 QTLs associated with volatile terpenes. To some extent, these results are in agreement with those obtained by GWAS for 85 carrot genotypes^23^. However, we were not able to confirm a QTL for the monoterpene ester bornyl acetate associated with the *DcTPS03* gene on chromosome 2. Instead, we identified a QTL for bornyl acetate on chromosome 1 that might be associated with the *DcTPS10* gene. We identified a large QTL cluster on chromosome 4 for sabinene, α-terpinene, γ-terpinene and terpinen-4-ol in leaves and 4-caren, sabinene and α-thujene in roots. QTLs associated with sabinene and terpinen-4-ol in roots have also been detected by a previous GWAS in the same genomic region^23^. A consistency of QTLs detected by QTL mapping with those identified by GWAS was observed in several crops such as soybean^36^, maize^37^, tea^38^ and rice^39^.

In silico analysis of the terpene synthase gene cluster on chromosome 4 revealed that it includes two zinc finger transcription factors, two methyltransferases, one phosphatase, one galacturonosyl transferase (GAUT), seven glycosyl hydrolases (GH) and five terpene synthases. A close association of terpene synthases with genes encoding enzymes that could modify terpenes or provide substrate for terpene synthases has already been described previously. For example, in *Arabidopsis thaliana*, gene groups including two to five terpene synthases clustered together with prenyltransferase-, cytochrome P450- and glycosyltransferase genes^40^. Similar results were obtained for tomato terpene synthase gene clusters on chromosomes 6, 7 and 8, which also contain several putative cytochromes P450, methyltransferases, acyltransferases and glycosyltransferases^41^. The location of these genes within or close to terpene synthase gene clusters might be beneficial for the inheritance of alleles from a single metabolic pathway and for the regulation of genes in the same biosynthetic pathway^42^. We found seven glycosyl hydrolases located within the terpene synthase gene cluster on chromosome 4. These enzymes are known to be involved in plant defence against pathogens and diverse physiological functions such as glycan biosynthesis, mobilization of energy, symbiosis, signalling and metabolism of glycolipids^43^. The role of glycosyl hydrolases in terpene metabolism remains to be investigated. A number of publications provide data suggesting a common evolution and functional specialization of terpene synthases and glycosyl hydrolases. A structural similarity of glycosyl hydrolases and monoterpene synthase has been shown for a limonene-type synthase of tobacco (5EAS). Two glycosyl hydrolase-like domains were found at its N-terminal region^44,45^. An early work showed that the GH activity resulted in the release of nerol, linalool, geraniol and terpineol in apricots^46^.

All five terpene synthase genes of the investigated gene cluster share a high amino acid sequence identity and belong to the TPS-b clade. They have the terpene synthase specific motives NSE/DTE and DDxxD that are essential for catalytic activity. These terpene synthases have the RR(x_8_)W motif at the N-terminus, which is characteristic for clade TPS-b. We predicted an N-terminal transit peptide for *Dc*TPS04, *Dc*TPS54, *Dc*TPS55 and *Dc*TPS27 suggesting their plastid localization. In contrast, *Dc*TPS26 that displays a high sequence similarity to other terpene synthases of the cluster lacks a proper transit peptide. This indicates a function as cytosolic terpene synthase^21^. Such a subcellular differentiation of closely related terpene synthases in the cytosol and plastids has been documented for (E,E)-β-farnesene synthase (*At*TPS03) and β-ocimene synthase (*At*TPS02) from *A. thaliana*^47^.

*DcTPS04* and *DcTPS54* were expressed differently in *yellow* and *cola* genotypes, which was consistent with the phenotypic data for volatile monoterpenes. Therefore, their enzymatic activity was investigated in an in vitro assay. The monoterpene synthase *Dc*TPS54 is a single-product sabinene synthase. *Dc*TPS04 catalysed the production of α-terpineol as a major product and four by-products including sabinene, β-limonene, β-pinene and myrcene. *Dc*TPS04 and *Dc*TPS54 share a sequence identity of 89.5%. They differ in several residues at the C-terminus that are reported to be critical for product profile^48^. *Dc*TPS04 has Gly and Asp at positions 589 and 594, respectively. *Dc*TPS54 has Ser at position 589 and Gln at position 594. Previous work on monoterpene synthases from Sitka spruce (*Picea sitchensis*) has shown that differences in individual amino acids may determine deviating product profile. Reciprocal mutagenesis analysis of a sabinene synthase and 3-carene synthase revealed that Phe at position 596 led to synthesis of sabinene, whereas its substitution to Leu directed product profile to 3-carene^48^. Sequence similarity and production outcome of *Dc*TPS04 and *Dc*TPS54 make them an attractive target for site-directed mutagenesis to identify, which particular structural feature of these enzymes affects their function.

Although we have provided several evidences (QTL and expression analysis, results of in vitro assay) suggesting the sabinene producing activity of Dc*TPS04* and Dc*TPS54*, the in vivo function of these TPSs needs to be proven conclusively.

Due to the high sequence identity, it could be suggested that the five terpene synthase genes have the same evolutionary origin through gene duplication. Terpene synthases associated with different products evolve by differential neofunctionalization and subfunctionalization. These evolutionary mechanisms are widely accepted for terpene synthases^21^. *Dc*TPS26 from the gene cluster on chromosome 4 lacks a transit peptide indicating its possible localization in cytosol and sesquiterpene synthase activity. Comparative functional characterization of *Dc*TPS26, *Dc*TPS04 and *Dc*TPS054 should be performed to confirm this hypothesis. Good examples of such functional divergence are the (E)-α-bergamotene synthase of the TPS-b clade from *Lavendula angustifolia* and the sesquiterpenes synthases *Sau*SesquiTPS of the TPS-a clade from *Santalum austrocaledonicum*^49,50^.

Sabinene appears to be among the major terpenes related to the preferred carrot flavour characteristics. However, it may also have negative impact due to its putative involvement in harsh and bitter notes. The information in the relevant literature is somewhat contradictory. Previous investigations suggested that sabinene is responsible for both “harsh” and “carrot top” flavour^13,15^. Another study showed that it might be also a major contributor to “fruity” flavour^16^. It may be assumed that the amount of sabinene is one of the critical factors for human gustatory sensation in terms of acceptance or rejection. Sensorial investigations based on large consumer panels and carrot cultivars selected by their variability for amounts of key volatile monoterpenes might be used to reveal the impact of single substances like sabinene.

Sabinene is not only one of the volatiles associated with carrot flavour and taste^11–13^ but also has ecological and economic relevance. For instance, it has been reported that sabinene can be involved in the plant defence against insects in Sitka spruce^7^. Besides its ecological benefits, sabinene is a major component of carrot seed oil^51^. Essential oil from wild carrot seeds possesses strong biological activity, probably due to the high content of sabinene and α-pinene. For instance, these monoterpenes were reported to be components of pharmaceuticals to treat the protozoan *Trypanosoma brucei* causing the African sleeping sickness disease^52^.

The results observed in this study are in good agreement with findings reported in a very recent paper describing functional characterization of carrot TPSs^53^ in the fully sequenced DH1 genotype^22^. This study also shows that the genomic region on chromosome 4 encodes TPSs catalysing the production of sabinene^53^. In complement to the findings of Muchlinski et al.^53^ we examined a sabinene-deficient carrot genotype. Taken together the complementary results of both studies are applicable in breeding programmes focused on modification of sabinene content in carrot. For this approach, we developed and validated a functional molecular marker that might serve as a useful breeding tool to select carrot accessions with high or low amounts of sabinene.

## Materials and methods

### Plant material and growth conditions

Two carrot mutants, *cola* and *yellow*, were involved in the experiments. The *cola* mutant possesses dwarfish morphology, whereas reduced amount of chlorophyll resulted in yellowish leaves of *yellow* mutant^54^. The F_2_ mapping population (VOM14) of 320 individuals was developed from an initial cross of a homozygous recessive *yellow* mutant as a female parent and a homozygous recessive *cola* mutant as pollen parent^54,55^. Plants were grown in 19 cm/30 cm w/h plastic pots in a sand/humus mixture (3/1) (v/v) under optimized greenhouse conditions at 25 °C/20 °C (day/night) and 18 h light photoperiod. They were drop irrigated and fertilized each week with 200 ml of a 0.3% Wuxal Super solution (8% N/8% P/6% K, Wilhelm Haug GmbH and Co.KG Düsseldorf, Germany).

### Volatile compound analysis of parent genotypes and F_2_ mapping population

The volatile compounds in root and leaf tissue of the parent genotypes and of the F_2_ mapping population were analysed by headspace SPME-gas chromatography as described previously^23^. Seven *yellow* and seven *cola* plants were involved in the initial parental phenotyping. Three hundred and twenty individual plants were investigated from the F_2_ mapping population.

### Marker-based QTL analysis

Genomic DNA was extracted from 0.5 g of young leaf tissue. The DNA concentration of samples was estimated by a Nanodrop 8000 (Thermo Scientific, Waltham, USA) and normalized to 10 ng μL^−1^. A universal fluorescent labelling strategy^56^ with M13 tailed forward primers was used to analyse SSRs and small InDels. The DNA fragments were separated on 6.5% polyacrylamide gels and detected by a LI-COR 4300 automatic sequencer (LI-COR Biosciences, Lincoln, NE, USA). The fragment sizes were calculated using fluorescent labelled 50–350 bp or 50–1500 bp size ladders, respectively. InDels and SNPs within or near to predicted terpene synthase (TPS) genes^23^ were detected by sequencing homologous PCR products of 800 bp to 1200 bp length of the *yellow* and *cola* parental lines (Tab. S3). SNPs within recognition sites of restriction endonucleases were converted to CAPS markers and analysed on 1.5% agarose gels. For other SNPs, KASP assays were developed (LGC Genomics, London, UK) and the marker analysis was performed using a CFX96 real-time cycler (Bio-Rad Laboratories, Hercules, USA). Amplification conditions were as follows: initial denaturation at 94 °C for 15 min; 10 cycles of 94 °C for 20 s, 61 °C for 1 min with a decrement of 0.6 °C/cycle followed by 29 cycles of 94 °C for 20 s, 55 °C for 1 min and final incubation for 10 min at 37 °C before plate reading. If necessary, additional three cycles with 55 °C annealing temperature (recycling) were performed. Data collection and allelic discrimination has been done with the Maestro^TM^ software (Bio-Rad Laboratories, Hercules, USA).

The obtained molecular marker data were analysed with JoinMap vers. 5.0 software (Kyazma B.V., Wageningen, The Netherlands)^57^. Loci were grouped using LOD thresholds from 2.0 to 6.0 in steps of 1.0 and recombination frequency lower than 0.4. The jump threshold was set to 5.0 and a third mapping round was not considered. The recombination frequencies were converted to mapping distances (in cM) using the Kosambi function. The linkage groups were assigned to the reference map using SSRs, SCARs and InDel markers with known chromosomal location^58^. QTL analysis was performed using the interval mapping (IM) mode of MapQTL vers. 5.0 software (Kyazma B.V., Wageningen, The Netherlands). Permutation tests (1000 permutations) were run for each root and leaf VOC to determine genome-wide significance thresholds (TH) (*P* = 0.05).

### Cloning of candidate genes

The *DcTPS04* (DCAR_013310) and *DcTPS54* (DCAR_013297) genes lacking the N-terminal transit peptide and the termination codons were amplified from carrot cDNA using gene-specific primers (Tab. S4), cloned into the pCR2.1-TOPO vector (Invitrogen/Thermo Scientific, Waltham, USA) and verified by sequencing. The *DcTPS04* and *DcTPS54* sequences were released from the pCR2.1-TOPO plasmid by *Nco*I and *Not*I restriction sites and ligated to identically digested pET28c expression vector (Novagen/Merck, Darmstadt, Germany) resulting in pET28c-*DcTPS04*-His and pET28c-*DcTPS54*-His constructs.

### In vitro enzyme assay and product identification by GC-MS

Following transformation of pET28c-*DcTPS04*-His and pET28c-*DcTPS54*-His plasmids in *E. coli* strain Rosetta^TM^ 2 (Novagen/Merck, Darmstadt, Germany), the individual colonies were inoculated in 100 mL LB medium supplemented with 50 mg l^−1^ kanamycin, cultivated at 37 °C until OD_600_ of 0.8, induced with 0.1 mM isopropylthio-β-galactoside (IPTG) and then grown for additional 16 h at 20 °C. Protein expression was verified by SDS-PAGE gel and Coomassie staining. Harvested bacterial pellets were resuspended in 5 ml assay buffer (250 mM HEPES-KOH buffer [pH 8], 100 mM MgCl_2_, 2.5 mM MnCl_2_, 50% glycerol, 1 mg µl^−1^ lysozyme), sonificated and collected by centrifugation. To perform the enzyme assay the crude bacteria extracts were mixed with 5 µl 1 M DTT, either 4 µl geranyl diphosphate (GPP, Sigma-Aldrich, St. Louis, USA) neryl diphosphate (NPP), *trans*-farnesyl diphosphate (E,E)-FPP and *cis*-farnesyl diphosphate (Z,Z)-FPP (Echelon Biosciences, Salt Lake City, USA) and filled with water to a final volume of 200 µl. After incubation at 41.5 °C for 0, 0.5, 1.5 and 4.5 h the terpene product was extracted with 200 µl hexane. For GC-MS analysis, an MPS2 autosampler was used (Gerstel, Mühlheim, Germany) and 1 µL of hexane extract was injected in splitless mode at 250 °C. Separation and detection of the products were performed by an Agilent Technologies 6890 GC equipped with a HP-INNOWax column (0.25 mm i.d., 30 m length, 0.5 μm film thickness) and an Agilent Technologies 5973N MS quadrupole analyser by electron ionization (source temperature: 230 °C, ionisation voltage: 70 eV). Carrier gas was helium with a flow rate of 1.0 mL min^−1^. Temperature programme: 40 °C (5 min), from 40 °C to 250 °C at 7 K min^−1^ and held 3 min at 250 °C, was used. Terpene synthase assay products were identified using authentic standards: myrcene, linalool, linalylisovalerat, 3-carene, α-terpineol, γ-terpinene, (+)-terpinen-4-ol, β-citronellol, β-limonene, sabinene, β-pinene, α-bisabolene, γ-bisabolene, (−)-α-bisabolol and α-farnesene (Sigma-Aldrich, St. Louis, USA).

### Expression analysis of terpene synthase genes in leaf and root tissue

For the expression analysis of candidate genes, we harvested leaf and root tissue from 16 week old carrot plants (*cola* and *yellow* mutants). The root samples are heterogeneous samples including cortex, xylem and phloem tissues. The leaf samples comprise of young leaf and petiole tissues. To reduce plant-specific differences, each sample was pooled from three individual plants, frozen in liquid nitrogen and ground to a fine powder. Total RNA was extracted from 100 mg tissue powder using the innuPREP Plant RNA Kit (Analytik Jena, Jena, Germany) according to the manufacturer’s instructions. RNA integrity was confirmed by agarose gel electrophoresis. Random hexamers were used as primers for first-strand cDNA synthesis with 2 µg total RNA as a template and Maxima First Strand cDNA Synthesis Kit (Thermo Scientific, Waltham, USA). Carrot tubulin 3α (*DcTUB*), heat shock 70 (*DcHSP*) and protein phosphatase 2 (*DcPP2A*) were used as internal reference genes. The expression levels of *DcTPS04*, *DcTPS26*, *DcTPS27*, *DcTPS54*, *DcTPS55*, *DcTUB, DcHSP* and *DcPP2A* genes were analysed with the qTOWER^3^ G touch (Analytic Jena, Jena, Germany) equipment and gene-specific primers (Tab. S4). The results were calculated using the Pfaffl method^59^.

### Reannotation of the carrot genome using GeMoMa 1.6.2beta

The published carrot genome was annotated using GeMoMa 1.6.2beta^22,28^. The 12 genomes (*Arabidopsis thaliana* (TAIR10), *Brachypodium distachyon* (v3.1), *Glycine max* (Wm82.a2.v1), *Lactuca sativa* (v5), *Mimulus guttatus* (v2.0), *Oryza savita* (v7.0), *Prunus persica* (v2.1), *Populus trichocarpa* (v3.1), *Sorghum bicolor* (v3.1.1), *Setaria italica* (v2.2), *Solanum lycopersicum* (v2.5) and *Theobroma cacao* (v1.1)) were used as reference with the software parameters: GeMoMa 1.6.2beta; SIMPLE PARAMETERS: reads: 1; splice: true; coverage: UNSTRANDED; gap opening: 11; gap extension: 1; maximum intron length: 15000; intron-loss-gain-penalty: 25; e-value: 100.0; contig threshold: 0.4; region threshold: 0.9; hit threshold: 0.9; predictions: 10; avoid stop: true; approx: true; prefix:; tag: prediction; verbose: false; timeout: 3600; sort: false; Score: ReAlign and GAF 1.6.2beta; SIMPLE PARAMETERS: tag: prediction; sorting: evidence, score; common border filter: 0.75; maximal number of transcripts per gene: 2147483647; prefix: AT; weight: 1.0; prefix: BD; weight: 1.0; prefix: GM; weight: 1.0; prefix: LS; weight: 1.0; prefix: MG; weight: 1.0; prefix: OS; weight: 1.0; prefix: PP; weight: 1.0; prefix: PT; weight: 1.0; prefix: SB; weight: 1.0; prefix: SI; weight: 1.0; prefix: SL; weight: 1.0; prefix: TC; weight: 1.0; filter: start==‘M’ and stop ==‘*’ and score/AA>=0.75; alternative transcript filter: tie==1 or evidence>1.

## Supplementary information

Tab S1. Identification of volatile terpenes

Supplemental material
