# Supplementary material for: The carrot monoterpene synthase gene cluster on chromosome 4 harbours genes encoding flavour-associated sabinene synthases
Source: Hortic Res. 2020 Dec 1;7:190. doi: 10.1038/s41438-020-00412-y (PMC7705728; doi:10.1038/s41438-020-00412-y)
Supplement: Supplementary file 2 — Supplemental material [file 41438_2020_412_MOESM2_ESM.docx]

**Supplemental Material:**


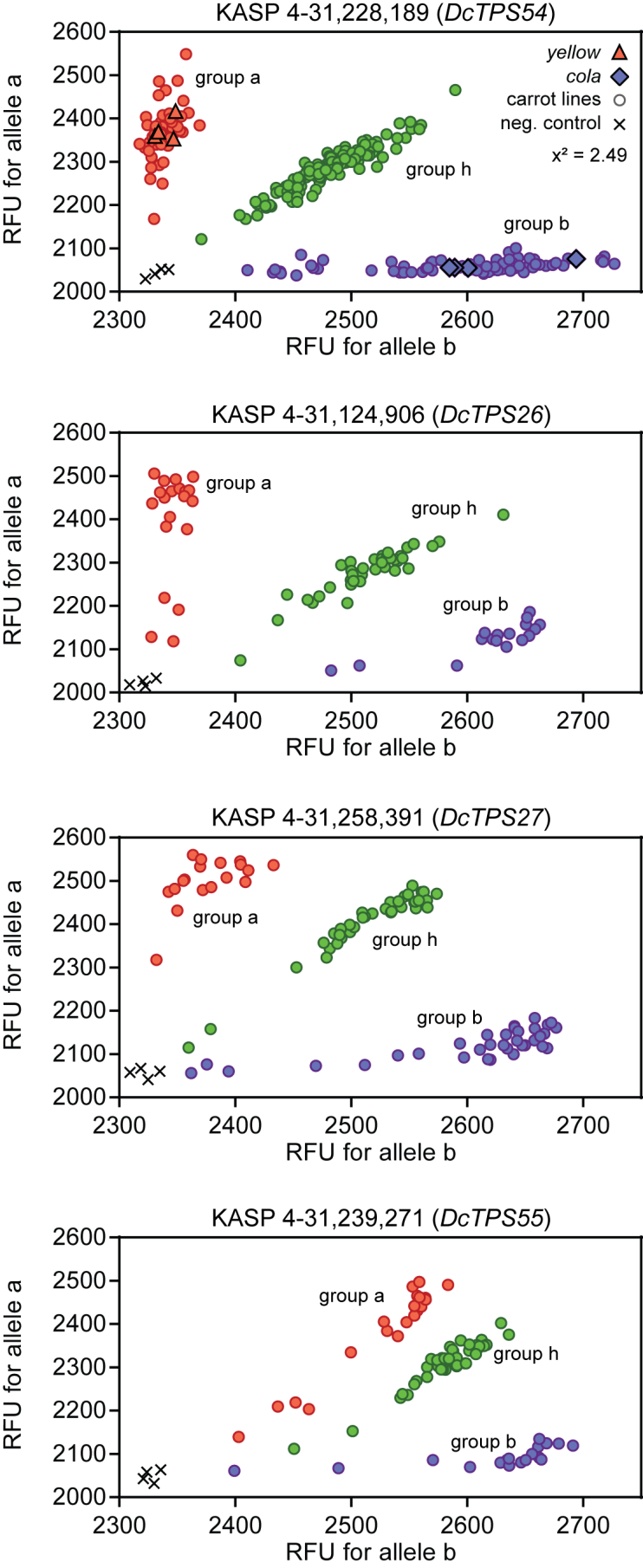


**Fig. S1: The genotypic analysis of the F_2_ biparental mapping population.** 320 individual plants were scored by KASP 4-31,228,189, KASP 4-31,124,906, KASP 4-31,258,391, KASP 4-31,239,271 molecular markers linked to *DcTPS54*, *DcTPS26*, *DcTPS27* and *DcTPS55* genes, respectively. The genotypes were discriminated in three groups, group a (red) carrying the homozygous maternal allele (a), in group b (blue) harbouring the homozygous paternal allele (b) and group h (green) with heterozygous plants. Data are shown as Relative Fluorescence Units (RFU).

**
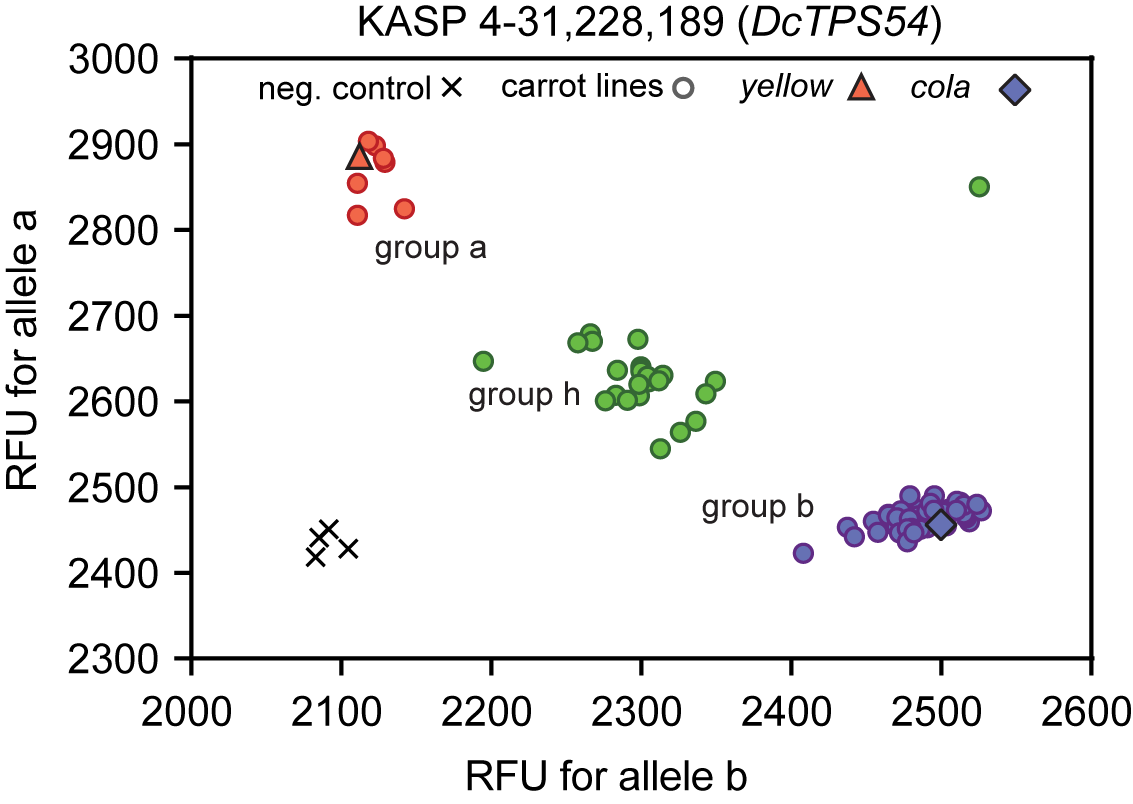
**

**Fig. S2: KASP genotyping of carrot accessions**. Eighty-five carrot genotypes were tested using the molecular marker KASP 4-31,228,189 linked to *DcTPS54* gene .Genotypic assignment was performed through reading the fluorescence emission of the HEX (allele a) and FAM (allele b) fluorophores for each sample in comparison to no template control. The plants in group a (red) carry the homozygous maternal allele (a), whereas the plants in group b (blue) harbour the homozygous paternal allele (b). Individuals in group h (blue) are the heterozygous plants. The results are shown as Relative Fluorescence Units (RFU).


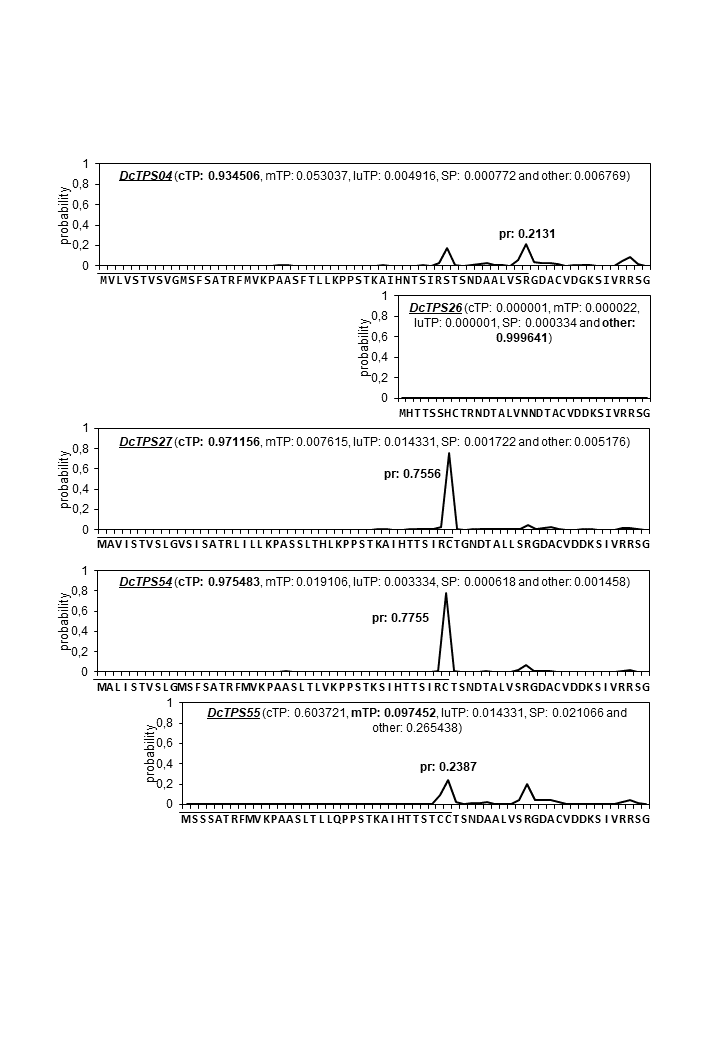


**Fig. S3: Prediction of the targeting peptides using Target P2.0** algorithm (Almagro Armenteros et al. 2019). Abbreviations are as follows: SP, signal peptide; mTP, mitochondrial transit peptide; cTP, chloroplast transit peptide; luTP, thylakoidal lumen composite transit peptide; other, no targeting peptide.


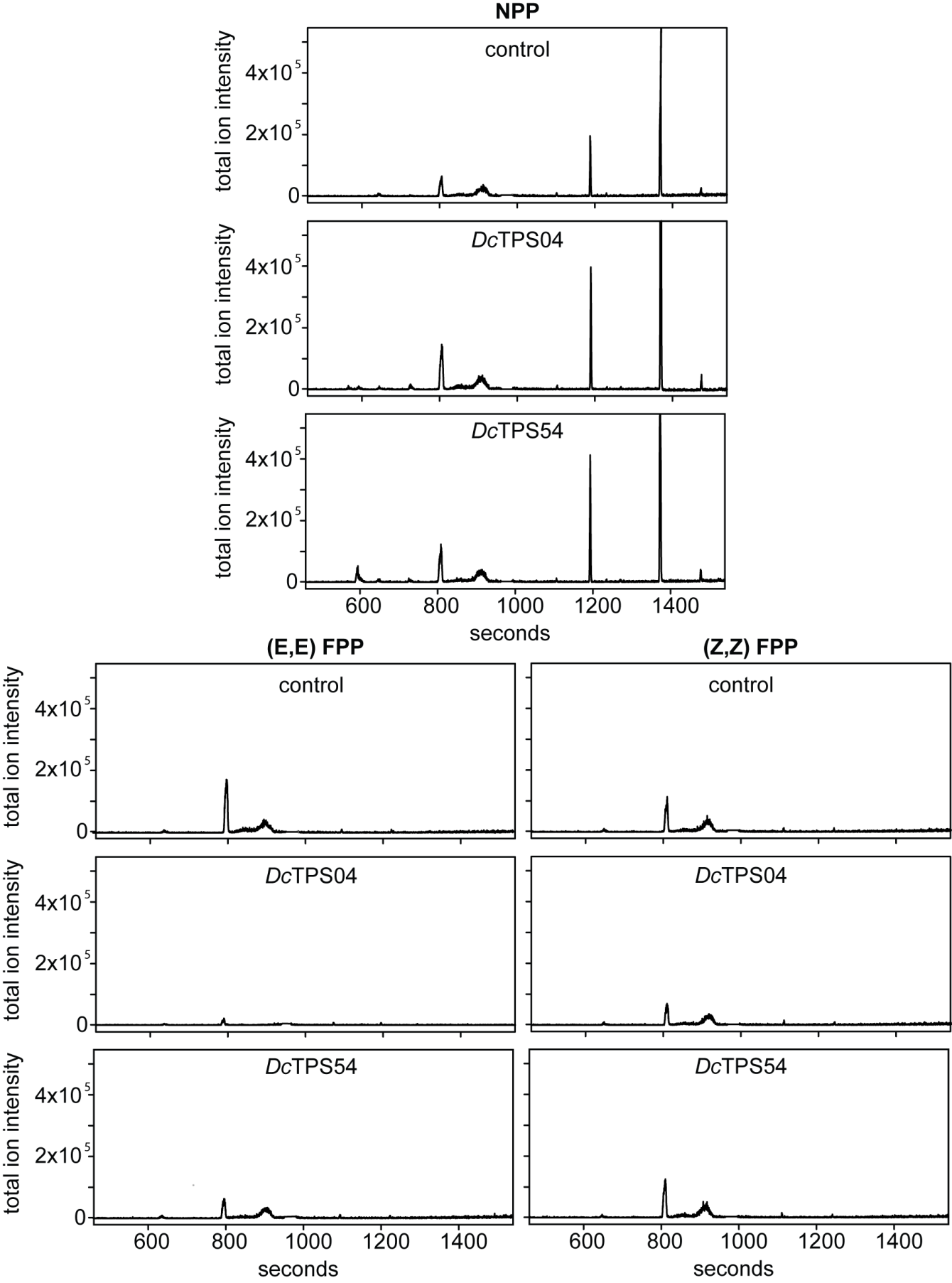


**Fig. S4**: GC-MS of the products formed by the Dc*TPS04* and Dc*TPS54* in an *in vitro* assay. The Dc*TPS04* and Dc*TPS54* proteins were incubated with NPP, (E,E)-FPP and (Z,Z)-FPP substrates for 30 minutes and products were analysed by GC-MS. The representative gas chromatograms for three individual experiments are shown.

**Tab S1. Identification of volatile terpenes** in the F_2_ biparental mapping population (Tab. S1 xlsx-file)

**Tab. S2: Molecular markers in or near to TPS genes used for the genotyping of the F_2_ mapping population (VOM14).**

| Marker | Type | Position | | |  |  | restriction |
| --- | --- | --- | --- | --- | --- | --- | --- |
|  |  | Chr. | Mb | to terpene synthase gene | **fw primer** | **rev primer** | enzyme |
| SSR_1-44,574,296 | SSR | 1 | 44,574,296 | 52,235 bp upstream of *DcTPS47* | ACCGGTGGAGAGCAATGTTA | TATGTGGGTGTGCGATTCCT |  |
| SSR_1-44,682,063 | SSR | 1 | 44,682,063 | Intron 4 of *DcTPS10* | AAGCTCCCCAGAAATCAGAA | TACCCCCGATGAACTTGAAC |  |
| CAPS_2-39,827,538 | CAPS | 2 | 39,827,538 | 228,499 bp downstream of *DcTPS03* | AGTGCCGTTTGAAATCTGCA | CCTGAATCCGCTCATTTGCA |  |
| InDel_3-45,438,469 | InDel | 3 | 45,438,469 | Intron 6 of *DcTPS05* | cacgacgttgtaaaacgacACTGGATTGACTTGGGAACG | CAAATCCAATCAAAAAGG |  |
| InDel_3-45,455,216 | InDel | 3 | 45,455,216 | 1,154 bp upstream of *DcTPS12* | GTTGGTCCAAATGCAAGGTT | TAAGGCCCAAAATGTCTTG |  |
| KASP_3-47,475,283 | KASP | 3 | 47,475,283 | 39 bp downstream of *DcTPS25* | gaaggtgaccaagttcatgctTATAGAGAAACTAGCAACGTCGCAA_(**first_fw)** gaaggtcggagtcaacggattATAGAGAAACTAGCAACGTCGCAT_(**second_fw)** | CCGTAGGATGTAACACGGAATAACAATAT |  |
| SSR_3-48,057,329 | SSR | 3 | 48,057,329 | 6,619 bp upstream of *DcTPS31* | CAAAACAACCCCACCTCACA | AAATCACCACGCCCTTTTCG |  |
| InDel_3-48,693,414 | InDel | 3 | 48,693,414 | Exon 3 of *DcTPS53* | TCGAATTTACCACCTCTCACCT | TTCGACCTCATGTCCTACAATG |  |
| CAPS_4-15,172,196 | CAPS | 4 | 15,172,196 | 324,930 bp upstream of *DcTPS38* | ATACGGAGAGGATGCAGAGC | ACGGCCTTAATTCCCTGTCA | *Sau*3AI |
| InDel+SSR_4-25,556,452 | InDel + SSR | 4 | 25,556,452 | 227 bp upstream of *DcTPS13* | GGTGGCCTGTCTACAAAAGT | CAGCCACAATCAGTCAGTGC |  |
| *DcTPS26*_4-31,147,953 | KASP | 4 | 31,147,953 | 563 bp downstream of *DcTPS26* | gaaggtgaccaagttcatgctCAAGTTTTCGTTTGTGTTTAGTCCTAAATTTT_(**first_fw)** gaaggtcggagtcaacggattAGTTTTCGTTTGTGTTTAGTCCTAAATTTC_(**second_fw)** | TATGGCAGTAACTTCTAAAACTCAGAAGAT |  |
| InDel_4-31,217,297 | InDel | 4 | 31,217,297 | 607 bp downstream of *DcTPS04* | cacgacgttgtaaaacgacCTGTGACAAAGCCTGTGGAA | GTTCACCCCATTCCTCTTGA |  |
| CAPS_4-31,218,832 | CAPS | 4 | 31,218,832 | Intron 3 of *DcTPS04* | CACAACCAAACATATGAGAGATTATG | GACACCATGTTCACGTTGGAAA | *Mse*I |
| InDel _4-31,227,503 | InDel | 4 | 31,227,503 | Exon 5 of *DcTPS54* | cacgacgttgtaaaacgacATGCTGCAAAATCCACATCA | AACCAAATTCGCTCTGAAGCAC |  |
| KASP_4-31,228,189 | KASP | 4 | 31,228,189 | Exon 3 of *DcTPS54* | gaaggtgaccaagttcatgctCACTGCTGTTAATGCCTTCATC_(**first_fw)** gaaggtcggagtcaacggattGCTCACTGCTGTTAATGCCTTCATA_(**second_fw)** | CCATASACATCATAGATATCGTCAATAGTA |  |
| KASP_4-31,239,271 | KASP | 4 | 31,239,271 | 5,188 bp upstream of *DcTPS55* | gaaggtgaccaagttcatgctAGTTAGTCAAGTGGCTAAGGTCTG**(first_fw)** gaaggtcggagtcaacggattATAGTTAGTCAAGTGGCTAAGGTCTA**(second_fw)** | GTGGCTGGATACTGACATGTTATTTGATT |  |
| KASP_4-31,258,147 | KASP | 4 | 31,258,147 | 6,155 bp downstream of *DcTPS27* | gaaggtcggagtcaacggattCTTACGATCAACGTGAATCAACCTAT_(**second_fw)** gaaggtcggagtcaacggattCTTACGATCAACGTGAATCAACCTAT_(**second_fw)** | GTCTAGATTAATATATTCGGCTCGAGCTA |  |
| CAPS_4-31,258,391 | CAPS | 4 | 31,258,391 | 6,399 bp downstream of *DcTPS27* | GAACACTGTTCTGATGGCCC | AACCCTCAGCCTTGACTCTC | *Hinf*I |
| SSR_5-8,195,539 | SSR | 5 | 8,195,539 | 56,668 bp upstream of *DcTPS56* | AGCTGTACTTTGGGGTGGACA | GTTCGTGCTGCTATTCCCAC |  |
| CAPS_5-20,491,579 | CAPS | 5 | 20,491,579 | 177,091 bp upstream of *DcTPS14* | CCCTACCGATGACATCACGA | AAACAACCACCTCCTTCGGC |  |
| InDel_5-29,660,476 | InDel | 5 | 29,660,476 | 3,718 bp upstream of *DcTPS57* | CTGATATTGGGGGTGTTTGG | CCCTCCCTCTTCTAAAAACCA |  |
| InDel_5-37,084,823 | InDel | 5 | 37,084,823 | 2,675 bp upstream of *DcTPS58* | GGGGAGTGGGAACTAGAACC | CATTTTCTCCCGGCATGCTT |  |
| InDel_5-37,091,591 | InDel | 5 | 37,091,591 | Exon 6 of *DcTPS33* | TCCAACGCCTCCACAGTTAT | AGGCTCCCTTATTCCGCTAC |  |
| SSR_6-1,253,007 | SSR | 6 | 1,253,007 | 201 bp downstream of *DcTPS01* | cacgacgttgtaaaacgacTGAGGATCTGTGCTCATTGC | CTTGCATGATCCAACTCCT |  |
| InDel_7-18,908,891 | InDel | 7 | 18,908,891 | 1,739 bp upstream of *DcTPS23* | TCTCGGACGTATGAACTCCA | GTCCAACAACGAAGACAGCA |  |
| SSR_8-17,405,472 | SSR | 8 | 17,405,472 | 24,608 bp upstream of *DcTPS29* | CAGGAAACGTCTGGATGCAG | CCGGCTTGTTTGGTTGGTAT |  |
| InDel_9-31,640,245 | InDel | 9 | 31,640,245 | 228,715 bp downstream of *DcTPS20* | ATGTGCAAGGAAATGCATCA | ATGCTGCACAAAGCATCAAG |  |
| KASP_9-31,870,687 | KASP | 9 | 31,870,687 | Exon 3 of *DcTPS20* | gaaggtgaccaagttcatgctAGGATTCATGTTACTGCTTCTTCCG_**(first_fw)** gaaggtcggagtcaacggattCAGGATTCATGTTACTGCTTCTTCCA_(**second_fw)** | CAAGATTAGAGGCCAGATGGTATATTGAT |  |

**Tab S3. The results of genotypic analysis for the F_2_ biparental mapping population**. Genotyping of 320 F_2_ plants was performed by CAPS 4-31,218,832 (Dc*TPS04*), KASP 4-31,124,906 (Dc*TPS26*), KASP 4-31,258,391 (Dc*TPS27*), KASP 4-31,228,189 (Dc*TPS54*) and KASP 4-31,239,271 (Dc*TPS55*) molecular markers. The plants in group a carry the homozygous maternal allele (a), whereas the plants in group b harbour the homozygous paternal allele (b). Individuals in group h are the heterozygous plants. In CAPS assay (CAPS 4-31,218,832 molecular marker), the amplified PCR products were restricted by *Mse*I resulting in different digestion pattern for allele a, b and heterozygous genotype h. In KASP assay (KASP 4-31,124,906, KASP 4-31,258,391, KASP 4-31,228,189 and KASP 4-31,239,271 molecular markers) the genotypes were discriminated through reading the fluorescence emission of the HEX (allele a) and FAM (allele b) fluorophores for each sample in comparison to no template control. ND: not determined.

| **molecular marker** | **ND** | **genotype** | | |
| --- | --- | --- | --- | --- |
|  |  | **a** | **h** | **b** |
| CAPS-4-31,218,832 | 29 | 70 | 160 | 61 |
| KASP-4-31,124,906 | 33 | 76 | 166 | 45 |
| KASP-4-31,258,391 | 37 | 75 | 153 | 55 |
| KASP-4-31,228,189 | 30 | 78 | 151 | 61 |
| KASP-4-31,239,271 | 132 | 52 | 98 | 38 |

**Tab. S4. Primers used in this study**. Primers used for cloning (C) of candidate terpene synthase genes. Primers used for expression analysis (EA) of terpene synthase genes. *Nco*I (CCATGG) and *Not*I (GCGGCCGC) restriction sites used for cloning are underlined. * indicates a primer combination published in ^53^.

| **Primer** | **Sequence (5'-3')** |  |
| --- | --- | --- |
| DcTPS04-194F | GGCCATGGTTCGTCGATCAGGCAATTATCC | C |
| DcTPS04-1822R | CCGCGGCCGCAAGAGGAATGGGGTGAACAAATAG | C |
| DcTPS54-194F | GGCCATGGTTCGTCGATCAGGCAATTATCC | C |
| DcTPS54-1822R | CCGCGGCCGCAAGAGGAATGGGGTGAACAAATAG | C |
| DcTPS04-172F | GTTGTCTACAGGAGCCGAAC | EA |
| DcTPS04-01R | CACTCTAATTAGCTAGTACATGCAAA | EA |
| DcTPS26-1779F | TTTGATTGGTGACCGAGACC | EA |
| DcTPS26-2000R | TGCTTACCAGGAGCAACTTA | EA |
| DcTPS27-1614F | GAATGTGCATGTTTACAATCATGTTT | EA |
| DcTPS27-1899R | AGATGATGCTGGTTTCAGCAAT | EA |
| DcTPS54-263F | TCAAGGAACGTGTGAGGATG | EA |
| DcTPS54-487R | ATCCACTGAAAGCCTGTTGC | EA |
| DcTPS55-1734F | AGGTTCTCGTGACAAAGAGC | EA |
| DcTPS55-1999R | ATGTGCACTGATGCGAAGAT | EA |
| DcTub-575F | CTCCTAAGGTTTCTGACACGG | EA |
| DcTub-738R | ATTCAAGTCTCCAAAGCTGGG | EA |
| DcHsp70_473F | GGAGATTCAACGACCCTTCC | EA |
| DcHsp70_656R | CCTAGAAACGCCTCTGCAAT | EA |
| *Dc*PP2A_F* | GTGTATCAATGTACCACCAGCAACT | EA |
| *Dc*PP2A_R* | GCTCACCAAGGAACATGACTTCTT | EA |
